# Transactions of Homœopathic Medical Society of New York for 1889

**Published:** 1890-05

**Authors:** 


### Transactions of the Homœopathic Medical Society of New York for 1889, and Transactions Homœopathic Medical Society of Pennsylvania for 1889.

We have received the *Transactions of the Homœopathic Medical Society of New York for* 1889, also *Transactions of the Homœopathic Medical Society of Pennsylvania for* 1889. Both volumes contain mixtures of allopathic and homœopathic treatment. In the last named we notice one gentleman, who calls himself a follower of Hahnemann, for uterine hemorrhage following placenta prævia, "gave every conceivable remedy indicated in such cases. Ergot in full doses had no effect. Finally, after he had given about ten drops of Bovinine to nourish the patient, he felt the uterus grow firmer, and on repeating the dose several times, he had the satisfaction to find the hemorrhage cease."

Query: If instead of giving "every conceivable remedy" he had followed Hahnemann's directions and given the *right* remedy, would he have needed Ergot or anything else? If only these people would use nothing more hurtful than Bovinine!

